# Depressive and anxiety symptoms among university students during the later stages of the COVID-19 pandemic in Germany - Results from the COVID 19 German Student Well-being Study (C19 GSWS)

**DOI:** 10.3389/fpubh.2024.1459501

**Published:** 2024-09-18

**Authors:** Eileen Heumann, Stefanie M. Helmer, Heide Busse, Sarah Negash, Johannes Horn, Claudia R. Pischke, Yasemin Niephaus, Christiane Stock

**Affiliations:** ^1^Institute of Health and Nursing Science, Corporate Member of Freie Universität Berlin and Humboldt-Universität zu Berlin, Charité – Universitätsmedizin Berlin, Berlin, Germany; ^2^Human and Health Sciences, Institute of Public Health and Nursing Science (IPP), University of Bremen, Bremen, Germany; ^3^Department Prevention and Evaluation, Leibniz Institute for Prevention Research and Epidemiology-BIPS, Bremen, Germany; ^4^Institute for Medical Epidemiology, Biometrics and Informatics, Interdisciplinary Center for Health Sciences, Medical School of the Martin Luther University Halle-Wittenberg, Halle, Germany; ^5^Institute of Medical Sociology, Centre for Health and Society, Medical Faculty, Heinrich-Heine-University Düsseldorf, Düsseldorf, Germany; ^6^Department of Social Sciences, University of Siegen, Siegen, Germany; ^7^Unit for Health Promotion Research, University of Southern Denmark, Esbjerg, Denmark

**Keywords:** anxiety, depression, mental health, university students, wellbeing

## Abstract

**Introduction:**

Data on the mental health of university students in Germany during the later stages of the pandemic is still limited. This study aimed to determine (1) the prevalence of anxiety and depressive symptoms among university students 1.5 years after the first COVID-19 restrictions and (2) which factors were associated with these outcomes.

**Methods:**

The cross-sectional COVID-19 German Student Well-being Study (C19 GSWS) collected data of 6,996 students at five German universities. Associations between anxiety and depressive symptoms with sociodemographic and other factors were analyzed using multivariable logistic regression models.

**Results:**

The mean age of the participants was 23.9 years (SD = 4.8), 67% were female and 31% male. The prevalence for depressive symptoms was 29 and 32% for anxiety. The lack of a trusted person and financial difficulties were associated with anxiety and depressive symptoms. Being worried that someone in one’s personal network had become severely ill with COVID-19 and concern about (re)infection with COVID-19 were associated with anxiety symptoms. Those with pre-existing health conditions had an up to 1.98-times higher chance for reporting depressive symptoms (OR, 95% CI: 1.01–3.88) and an up to 2.27-times higher chance for anxiety symptoms, respectively (OR, 95% CI: 1.15–4.46).

**Conclusion:**

Concepts for prevention and counseling to tackle mental health problems in students are needed and programs should take specific stressors in times of crises into account.

## 1 Introduction

Mental health problems, such as depressive and anxiety symptoms, are widespread among university students worldwide (1, 2) and in Germany (3, 4). In the World Mental Health Survey, Auerbach, Alonso (2) found that one-fifth (20.3%) of university students aged 18–22 years in 21 countries indicated the presence of a mental health disorder (including e. g. anxiety or depressive disorders). Similarly, in Germany, a recent systematic review with meta-analysis indicates that around one in five students is affected by depressive symptoms (4). This pooled prevalence rate (21.1%) is more than twice as high as the elevated prevalence rate of depressive symptoms observed in the general population in Germany during the Corona Virus Disease 2019 (COVID-19) pandemic (5).

Mental health problems in university students are associated with low academic attainment (6) and elevated risk for university dropout (7, 8). Further associations are described with substance use (9, 10), unsafe sexual behavior, low levels of physical activity and poor physical health status (11). Such behaviors can serve as coping mechanisms for underlying mental health issues, but they often exacerbate the problems, creating a vicious cycle that further impairs students’ wellbeing and academic success (12). Moreover, research has shown correlations between depressive symptoms in university students and low quality of life, self-injurious (10), and suicidal behavior (9, 13).

Reasons for poor mental health among university students may include the presence of stressors at individual, interpersonal, or systemic levels (14, 15) of which some are modifiable, and some are not (15, 16). Among the non-modifiable risk factors, female students (16), and sexual and gender minorities (9, 17) are at high risk. Among the modifiable factors, little social support (18), financial constraints (19, 20) and low socio-economic status (15), interpersonal stressors (e.g., romantic and peer relationships) (16, 21) or pre-existing health problems (16) are considered to be associated with depressive symptoms. The occurrence of mental health symptoms is multifaceted and influenced by various factors (14–16). According to the socio-ecological model, these factors operate on both individual and broader, supra-individual levels, including, e.g., interpersonal relationships and the environments in which individuals interact (22).

The COVID-19 pandemic produced additional stressors in the daily lives of university students (23). As in many other countries, restrictions to limit the spread of the virus were in place in Germany, in general, and at university level. Quantitative data showed that the pandemic-associated protection measures, such as the switch from face-to-face to online teaching and the cancelation of internships and other practice-based teaching and associated life changes, had an impact on university students’ mental health (24, 25).

Consequences for students’ mental health during the COVID-19 pandemic included, among others, anxiety, stress, worry, and depressive symptoms (14, 21, 26, 27). Evidence showed exacerbated trends in depressive and anxiety symptoms among university students (21). Cross-national studies conducted during the early phase of the COVID-19 pandemic found the prevalence of depressive symptoms in university students to differ between European countries (28), with German students presenting higher rates of depressive symptoms compared to Northern European university students (e. g. Sweden, Denmark, Norway) and lower rates compared to Southern European university students (e. g. Italy, Portugal, Spain) (27).

According to Cao, Fang (26), 25% of university students were experiencing anxiety symptoms associated with academic concerns, financial worries, and the impact of COVID-19 on their daily life. A study based on data from German university students conducted during the first COVID-19-related lockdown in spring 2020 indicated an association between elevated academic stress, dissatisfaction with university and depressive symptoms (25). Additionally, a high proportion of university students reported that they were unable to effectively cope with the stress associated with the pandemic situation (29).

Numerous studies examining the mental wellbeing of university students during the course of the pandemic were predominantly carried out in China (21, 30) and other countries (29). However, the availability of data in Germany is limited and most of the data was collected at an early stage of the pandemic [e.g., (25, 31–33)]. Further, longitudinal studies in the United States indicated a potential causal relationship between the pandemic and mental health outcomes (34, 35).

Given the dearth of data and the persistent stressors associated with the pandemic, it remains critical to continuously monitor the mental wellbeing of university students in Germany and contribute to the existing database. Thus, this study aimed to investigate the mental wellbeing of university students in Germany during a later stage of the COVID-19 pandemic. The objectives were to assess (1) levels of depressive and anxiety symptoms among university students and (2) factors associated with these outcomes.

## 2 Methods

### 2.1 Study design

The German COVID-19 Student Wellbeing Study (C19 GSWS) is a cross-sectional study in which an online questionnaire was implemented at five universities in Germany (Charité – Universitätsmedizin Berlin, University of Bremen, University of Siegen, Martin Luther University Halle-Wittenberg, and Heinrich-Heine-University Düsseldorf) in a later phase of the pandemic. The study is based on the COVID-19 International Student Wellbeing Study (C19 ISWS) that was conducted during the early phase of the COVID-19 pandemic in spring 2020 in 27 European countries (28).

### 2.2 Data collection and context

The data collection was carried out using LimeSurvey between October 27 and November 14, 2021, at each of the participating universities. During the survey period, several COVID-19 regulations, such as the obligation of wearing masks indoors or hygiene measures were still in place. At this time, the incidence of infection increased a fourth time, driven by the spread of the SARS-CoV-2 type delta. Therefore, some German universities decided not to return to face-to-face teaching at all and to teach remotely throughout the whole winter semester, whereas others offered only a few face-to-face courses in smaller learning groups. Charité – Universitätsmedizin Berlin, for example, as a university hospital, opted for predominantly remote learning to minimize in-person contact due to high infection rates and stringent safety regulations. At Martin Luther University Halle-Wittenberg a limited number of face-to-face interactions, mainly in smaller groups and under strict health guidelines were allowed. Accordingly, the learning and teaching situation at German universities varied substantially at the time of the data collection but was different from the normal situation at all universities.

The questionnaire used was based on the original questionnaire of the C19 ISWS and was slightly modified considering changed circumstances such as the availability of vaccines. The modified questionnaire included questions on socio-demographic factors, health behavior, health condition, mental wellbeing, financial resources, perceived study conditions during the pandemic, critical health literacy, vaccination status against coronavirus, and attitudes toward COVID-19 vaccinations.

### 2.3 Recruitment and participation

University students aged 18 and above who were currently enrolled as students in undergraduate, graduate, or doctoral programs were invited to participate in the study. Since participants entered the study through self-selection, convenience sampling was employed. University students were invited via email, as well as through e-learning platforms (Martin Luther University Halle-Wittenberg and University of Bremen). At Heinrich-Heine-University Düsseldorf invitations were also distributed via Instagram. University students were given the option to take the survey in German or English. All participants provided their informed consent before completing the survey. Ethical approval was obtained from the ethics committees of each of the five participating universities.

### 2.4 Measures

#### 2.4.1 Subjective depressive and anxiety symptoms

Subjective depressive symptoms were assessed using the Centre for Epidemiological Studies Depression Scale with eight items (CES-D 8) (36) and a short-form version of the Patient Health Questionnaire (PHQ-2 scale) (37, 38). Also, an abbreviated form of the Generalized Anxiety Disorder scale (GAD-2) was used to assess anxiety symptoms (39).

The CES-D 8 was used to assess the frequency and severity of subjective depressive symptoms (36). University students were asked how often during the last week (1) they felt depressed, (2) everything was an effort, (3) they slept restlessly, (4) could not get going, (5) felt lonely or sad, or (6) they enjoyed life and felt happy (last two items were reverse coded items). Responses ranged from (0) ‘none or almost none of the time’; (1) ‘some of the time’; (2) ‘most of the time’ to (3) ‘all or almost all of the time’ on a four-point Likert scale. Summarizing all items resulted in a continuous non-weighted CES-D 8 score, with a higher rating indicating a higher level of depressive symptoms (score ranging from 0 to 24). Since there is no validated cut-off point for the CES-D 8 to estimate the prevalence of depressive symptoms in this population group, we report the mean value and conduct further analyses using the PHQ-2 and GAD-2 only.

The PHQ-2 consists of the first two items of the PHQ-9 (38, 40). The master question is: ‘Over the last 2 weeks, how often have you been bothered by the following problems?’ The two items are ‘feeling down, depressed or hopeless’ and ‘little interest or pleasure in things’. For each item, the response options on a four-point Likert scale are (0) ‘not at all’, (1) ‘several days’, (2) ‘more than half the days’, and (3) ‘nearly every day’. After summarizing the items, the overall PHQ-2 score can range from 0 to 6 (38). For analysis, we used a cut-off point of 3 as suggested based on the available literature (37) to indicate whether the participants showed relevant depressive symptoms or not (0 to 3 ‘no depressive symptoms’; 4 to 6 ‘depressive symptoms’). Using the same master question with the same scaling and cut-off, the GAD-2 was conducted with the following items: ‘feeling nervous, anxious, or on edge’ and ‘not being able to stop or control worrying’ (39, 41). The PHQ-2 and GAD-2 can be combined to form the PHQ-4, a concise tool that simultaneously screens for symptoms of depression and anxiety in just four questions. This combination allows for an efficient assessment of both mental health conditions (42, 43).

The PHQ-2 and GAD-2 are reliable and validated for the university context (44, 45) as well as the long version of the CES-D (46). Nevertheless, the short version of the CES-D is commonly used within this population group and also in the previous C19 ISWS (28). The Cronbach’s alpha in our sample was 0.86 for CES-D 8, 0.79 for PHQ-2, and 0.78 for GAD-2.

### 2.5 Covariates

#### 2.5.1 Socio-demographic factors

The following information on the socio-demographic characteristics was assessed for this investigation: age [‘between 18 and 25 years old’ (ref.) vs. ‘aged 26 and older’], gender [‘male’/‘female’ (ref.)/‘diverse’], relationship status [‘single’/‘in a relationship’ (ref.)/‘it is complicated’], migrant background [‘no migrant background’ (ref.)/‘one parent born outside Germany’/‘both parents born outside Germany’], as well as a place of birth [‘Germany’ (ref.) vs. ‘other’], residence status in Germany [‘permanent residency’ (ref.) vs. ‘temporary residency’] and housing situation [‘living alone’ vs. ‘living with other persons in the household’ (ref.)]. Age was dichotomized as suggested by Van de Velde, Buffel (27).

#### 2.5.2 Socio-economic and social support factors

As university students have not completed their educational training, yet, and their actual income or employment status is not adequate for assessing their socio-economic status, following Van de Velde, Buffel (27), the highest level of education of each parent [‘less than secondary’/‘secondary’/‘higher education’ (ref.)], was used as an indicator of their socio-economic status. For university students’ current subjective financial status, they were asked to report to what extent they agreed with the statement ‘I have sufficient financial resources to cover my monthly costs’. Those who (strongly) agreed with this statement were grouped (ref.) and distinguished from those who (strongly) disagreed. Again, following Van de Velde, Buffel (27) participants were asked from how many people within their network (partner, parents, siblings, grandparents, friends, colleagues, and/or acquaintances) they could easily borrow 500 euros within 2 days [‘zero’/‘one to two’/‘three to four’/‘five or more persons’ (ref.)] to assess their social and economic capital. Lastly, the extent of social support was measured by assessing the availability of a trusted person with whom to discuss intimate matters [‘yes’ (ref.) vs. ‘no’].

#### 2.5.3 Study-related factors

University students were asked which degree program they were enrolled in [‘Bachelor’s degree program’ (ref.)/‘Master’s degree program’/‘State examination (medicine, law)’]. Students with unspecific or without information on their degree program as well as doctoral students were excluded (total 242 persons). Doctoral students in Germany are not comparable to other student groups because they often have distinct roles, such as contributing to university research and teaching, which differ from typical student responsibilities. Moreover, it was assessed whether university students were in the first year or in a higher semester (ref.). The field of study was dichotomized for the analysis [‘health-related degree program’ (ref.) vs. ‘other’].

#### 2.5.4 Health-related factors

Further health-related variables were included for more in-depth analyses including pre-existing diseases. University students could select several pre-existing conditions in the questionnaire. For the analysis, we kept a nominal variable with seven values [‘no pre-existing health condition’ (ref.)/‘metabolic disease/‘cardiovascular disease’/‘lung disease’/‘obesity’/immunosuppressed disease/‘two or more pre-existing health conditions’].

COVID-19-related factors were also included, such as whether or not participants currently have or had COVID-19 disease. If so, they were asked to indicate on an 11-point numeric rating scale how worried they were that (1) they would get infected with the coronavirus one more time and that (2) they would get seriously ill from a new infection (0 = ‘not worried at all’, 10 = ‘very worried’). Participants who had previously stated that they did not have COVID-19 disease were asked how worried they were about (1) becoming infected with the virus and (2) becoming severely ill from infection (0 = ‘not at all worried’, 10 = ‘very worried’). University students were also asked how worried they were that someone from their personal network would (1) become infected with COVID-19 or (2) become seriously ill from an infection. Finally, concerns were assessed about medical staff and hospitals not being adequately equipped to deal with the pandemic, as well as confidence about whether the necessary medical support can be obtained in case of COVID-19 disease. All COVID-19-related variables were dichotomized for analysis [‘not at all” to ‘little concerned’ (ref.) vs. ‘fairly to very concerned’ or’ not at all to little optimistic’ vs. ‘fairly to very optimistic’ (ref.)].

### 2.6 Data analysis

Descriptive statistics were calculated to summarize the sample in terms of socio-demographic data and study-related information. Prevalence rates were calculated for depressive and anxiety symptoms during the COVID-19 pandemic using the respective cut-off values (PHQ-2 and GAD-2).

Two multivariable logistic regressions for each mental health outcome were carried out to determine the associations with selected independent variables. In the first model, socio-demographic, socio-economic, and social support factors, as well as study-related factors, were considered. In the second model, health-related factors and COVID-19-related stressors were also included. The analyses were based upon a prior analysis conducted on the C19 ISWS dataset during the first phase of the pandemic (27). Before entering the independent variables into the model, multicollinearity between independent variables was assessed based on tolerance and VIF coefficients. Indices for the second regression model indicated that a multicollinearity problem occurred, which was solved by removing one COVID-19 related variable (‘How worried are you that you will get severely ill from a COVID-19 infection?’). The data analysis was conducted using IBM SPSS version 28 and the complete data set can be retrieved from Zenodo (47).

## 3 Results

### 3.1 Sample

After data cleaning 6,996 cases remained for data analysis. Somewhat less than a third of the participants came from the Martin-Luther-University Halle-Wittenberg (29.8%, n = 2,087), about a quarter from the University of Bremen (25.3%, *n* = 1,771), a fifth from the University of Siegen (22.0%, *n* = 1,540), 15.5% (*n* = 1,084) from Charité – Universitätsmedizin Berlin and 7.3% (*n* = 514) from Heinrich-Heine-University Düsseldorf. Response rates ranged between 7.8% (University of Bremen) and 13.3% (Charité – Universitätsmedizin Berlin).

Most participants were between 20 and 23 years old (48.3%), the mean age was 23.9 years (SD = 4.8; Min = 18; Max = 68). More than two-thirds of the participants were female (67.3%). Almost half of the respondents were studying for a bachelor’s degree (47.2%). The study sample is described in further detail in Table 1.

**Table 1:** Socio-demographic characteristics of the sample.

	*n*	%
Age		
<20	666	9.5
20–23	3,368	48.3
24–27	1,851	26.5
28–30	511	7.3
≥ 31	580	8.3
Gender*		
Male	2,121	30.4
Female	4,702	67.3
Diverse	74	1.1
Degree program		
Bachelor program	3,305	47.2
Master program	1,385	19.8
State examination (medicine, law)	2,306	33.0
Relationship status*		
In a steady relationship	3,667	52.5
Single	2,900	41.5
It is complicated	292	4.2
Living situation		
Alone	1,418	20.9
With others	5,373	79.1
Person to discuss intimate matters with		
Yes	5,529	90.4
No	590	9.6
Financial resources		
Sufficient to cover monthly costs	5,424	77.8
Neither nor	574	8.2
Not sufficient to cover monthly costs	974	14.0
Status in Germany		
Permanent residency	6,764	97.2
Temporary residency	196	2.8

*Missing percentages due to answer options ‘no information’ or ‘I do not know’.

### 3.2 Depressive and anxiety symptoms

According to the PHQ-2, 28.9% of participants reported depressive symptoms. Female university students were more affected by depressive symptoms than male university students (29.6% compared to 26.2%). Among people of diverse gender, the prevalence rate was 43.1%. An analysis of the CES-D 8 showed an average score of 9.42 points (SD = 4.85).

The GAD-2 revealed that 31.5% of the sample reported anxiety symptoms, whereas the gender-specific prevalence again differed: 34.0% of female university students reported anxiety symptoms, while among male university students it was only 24.1%. People with diverse gender identity stated that they were distinctly more often affected by anxiety (63.9%).

### 3.3 Model 1

Considering associations between socio-demographic, socio-economic, social support factors, as well as study-related factors and wellbeing outcomes, the results of model 1 (Table 2) showed that male compared to female university students, master’s compared to bachelor’s students, first-year compared to advanced university students, as well as those of health-related subjects compared to university students of other subjects, experienced fewer depressive symptoms. On the other side, having a complicated rather than a steady relationship, not having a trusted person, and living alone compared instead of living with others were factors that were significantly associated with more frequent depressive symptoms. Moreover, financial issues were found to be associated with depressive symptoms: e.g., university students who were not able to borrow money from anyone as well as those who did not have enough money to cover their monthly expenses reported more depressive symptoms.

**Table 2:** Associations between socio-demographic, socio-economic, social support factors, study-related factors and depressive symptoms as well as anxiety: results of two binary logistic regressions (model 1).

		Depressive symptoms (PHQ-2, *n* = 4,963)		Anxiety symptoms (GAD-2, *n* = 4,962)	
Variables					
		OR	95%-CI	OR	95%-CI
Age	18–25	1.00		1.00	
	≥26	0.91	0.77–1.09	1.02	0.86–1.20
Gender	Female	1.00		1.00	
	Male	**0.71**	**0.61–0.83**	**0.54**	**0.46–0.63**
	Diverse	1.22	0.65–2.28	**3.32**	**1.77–6.26**
Migration background	No migration background	1.00		1.00	
	One parent born outside Germany	1.23	0.97–1.57	**1.33**	**1.06–1.68**
	Both parents born outside Germany	1.26	0.97–1.64	**1.37**	**1.06–1.78**
Place of birth	Germany	1.00		1.00	
	Outside Germany	1.05	0.73–1.53	0.86	0.60–1.24
Relationship status	Relationship	1.00		1.00	
	Single	1.01	0.87–1.16	1.06	0.92–1.22
	Complicated	**1.58**	**1.17–2.15**	**1.47**	**1.08–1.98**
Residency status	Permanent residency	1.00		1.00	
	Temporary residency	0.97	0.57–1.64	1.06	0.64–1.77
Housing situation	Living with others	1.00		1.00	
	Living alone	**1.18**	**1.00** ^**a**^ **-1.39**	1.06	0.91-1.25
Level of education (Mother)	Higher education	1.00		1.00	
	Secondary	0.92	0.76–1.11	1.00	0.83–1.19
	Less than secondary	0.98	0.83–1.17	0.96	0.81–1.14
Level of education (Father)	Higher education	1.00		1.00	
	Secondary	1.05	0.86–1.27	0.96	0.79–1.16
	Less than secondary	1.09	0.92–1.30	1.02	0.86–1.21
Financial situation	Sufficient financial resources	1.00		1.00	
	Insufficient financial resources	**2.20**	**1.84–2.63**	**1.92**	**1.61–2.29**
Possibility of borrowing money	≥ 5 persons	1.00		1.00	
	3–4 persons	**1.37**	**1.17–1.61**	**1.57**	**1.35–1.83**
	1–2 persons	**1.57**	**1.30–1.88**	**1.85**	**1.55–2.20**
	0 persons	**2.18**	**1.60–2.97**	**2.24**	**1.65–3.04**
Person to trust	Yes	1.00		1.00	
	No	**3.44**	**2.77–4.28**	**2.66**	**2.14–3.30**
Degree program	Bachelor	1.00		1.00	
	Master	**0.83**	**0.69–0.99**	0.89	0.74–1.06
	State examination	0.89	0.73–1.07	**0.80**	**0.67–0.96**
Year of study	≥ 2 semesters	1.00		1.00	
	First year	**0.76**	**0.64–0.91**	0.98	0.83–1.16
Field of study	Other	1.00		1.00	
	Health-related	**0.63**	**0.52–0.77**	**0.77**	**0.64–0.93**

^a^Due to rounding of the results. Statistically significant results are printed in bold.

Concerning anxiety, male gender, and a health-related degree program were protective factors compared to being female and student in other degree programs, respectively. People of diverse gender (as compared to female university students), those with a complicated relationship status, without a trusted person or in a difficult financial situation (i.e., without the possibility of borrowing money from someone) and difficulty covering monthly expenses had a higher chance of experiencing anxiety. The migration background was also negatively associated with anxiety (i.e., both parents born outside Germany compared to both parents born in Germany).

### 3.4 Model 2

For model 2, health-related variables were added to model 1 (Table 3). This second model showed that university students with pre-existing conditions reported more mental health problems compared to those without underlying health conditions: Suffering from cardiovascular disease or multiple diseases was associated with an increased likelihood for reporting depressive symptoms. A pre-existing cardiovascular disease and obesity were associated with anxiety symptoms. Metabolic health conditions seemed to be a protective factor for anxiety symptoms. Regarding the COVID-19-related factors, worry that someone from the personal network got severely ill with COVID-19 and concern about (re-)infection with COVID-19 was associated with anxiety symptoms. Being concerned about (re-)infection with COVID-19 was also associated with depressive symptoms.

Being a first-year university student, being enrolled in a health-related program, and identifying as male appeared to serve as protective factors against depressive symptoms. Experiencing financial difficulties, being in a complicated relationship, and lack of social support were associated with higher levels of depressive symptoms. Similarly, in the initial model, male gender and enrolment in health-related subjects were identified as protective factors in regard to anxiety symptoms. Conversely, diverse gender identity, lacking a trusted person, financial constraints, and a migration background (both parents) were all negatively associated with anxiety symptoms (Table 3).

**Table 3:** Associations between socio-demographic, socio-economic, and social support factors, study-, health-and COVID-related factors and depressive symptoms and anxiety: results of two binary logistic regressions (model 2).

		**Depressive symptoms** (PHQ-2, *n* = 2,303)		**Anxiety** (GAD-2, *n* = 2,303)	
Variables					
		OR	95%-CI	OR	95%-CI
Age	18–25	1.00		1.00	
	≥ 26	0.78	0.60–1.01	0.90	0.69–1.14
Gender	Female	1.00		1.00	
	Male	**0.78**	**0.62–0.99**	**0.62**	**0.49–0.78**
	Diverse	1.41	0.59–3.36	**3.91**	**1.59–9.59**
Migration background	No migration background	1.00		1.00	
	One parent born outside Germany	1.12	0.79–1.59	1.23	0.87–1.73
	Both parents born outside Germany	1.21	0.80–1.83	**1.87**	**1.25–2.78**
Place of birth	Germany	1.00		1.00	
	Outside Germany	1.01	0.55–1.84	0.81	0.46–1.45
Relationship status	Relationship	1.00		1.00	
	Single	1.05	0.85–1.30	1.23	1.00^a^-1.51
	Complicated	**1.79**	**1.12–2.86**	1.57	0.98–2.53
Residency status	Permanent residency	1.00		1.00	
	Temporary residency	0.69	0.29–1.64	0.70	0.31–1.57
Housing situation	Living with others	1.00		1.00	
	Living alone	1.07	0.83–1.37	0.91	0.72–1.16
Level of education (Mother)	Higher education	1.00		1.00	
	Secondary	0.99	0.75–1.31	0.99	0.75–1.29
	Less than secondary	1.11	0.86–1.45	0.91	0.71–1.18
Level of education (Father)	Higher education	1.00		1.00	
	Secondary	0.99	0.77–1.28	0.98	0.73–1.30
	Less than secondary	1.07	0.80–1.43	0.98	0.77–1.26
Financial situation	Sufficient financial resources	1.00		1.00	
	Insufficient financial resources	**1.91**	**1.45–2.52**	**1.70**	**1.29–2.24**
Possibility of borrowing money	≥ 5 persons	1.00		1.00	
	3–4 persons	**1.48**	**1.17–1.86**	**1.73**	**1.38–2.17**
	1–2 persons	**1.40**	**1.06–1.84**	**1.91**	**1.47–2.49**
	0 persons	**2.07**	**1.25–3.42**	**2.10**	**1.28–3.45**
Person to trust	Yes	1.00		1.00	
	No	**3.89**	**2.76–5.48**	**2.63**	**1.87–3.71**
Degree program	Bachelor	1.00		1.00	
	Master	0.86	0.65–1.12	1.01	0.78–1.32
	State examination	0.90	0.68–1.18	0.87	0.67–1.14
Year of study	≥ 2 semesters	1.00		1.00	
	First year	**0.73**	**0.56–0.96**	1.01	0.79–1.30
Field of study	Other	1.00		1.00	
	Health-related	**0.63**	**0.47–0.84**	**0.69**	**0.52–0.91**
Pre-existing disease	None	1.00		1.00	
	Metabolic disease	0.38	0.10–1.42	**0.20**	**0.04–0.94**
	Cardiovascular disease	**1.98**	**1.01–3.88**	**2.27**	**1.15–4.46**
	Lung disease	0.86	0.52–1.43	0.94	0.58–1.53
	Obesity	1.59	0.99–2.57	**1.61**	**1.00** ^**a**^ **-2.57**
	Immunocompromised/miscellaneous	0.91	0.39–2.11	1.03	0.46–2.28
	Multiple diseases	**1.87**	**1.06–3.28**	1.34	0.76–2.36
COVID infection	No	1.00		1.00	
	Yes	1.14	0.78–1.67	1.14	0.79–1.64
Concern to get infected	No	1.00		1.00	
	Yes	**1.31**	**1.04–1.65**	**1.37**	**1.09–1.71**
Concern relatives get infected	Yes	1.00		1.00	
	No	0.93	0.65–1.31	1.25	0.89–1.76
Concern relatives get severely ill	Yes	1.00		1.00	
	No	0.77	0.55–1.08	**1.56**	**1.12–2.16**
Confidence in receive medical care in case of infection	Yes	1.00		1.00	
	No	1.38	0.95–1.99	1.36	0.95–1.96
Concern doctors/hospitals do not have adequate supplies	Yes	1.00		1.00	
	No	0.90	0.72–1.12	0.85	0.69–1.06

Statistically significant results are printed in bold.

## 4 Discussion

Our study aimed at assessing the prevalence of anxiety and depressive symptoms in a later stage of the COVID-19 pandemic among German university students. The prevalence of depressive symptoms within this sample was 28.9%, which is in line with other studies conducted during the COVID-19 pandemic in similar populations in Germany (48, 49) and worldwide (30, 50). The calculated mean value of the CES-D 8 in this sample is with 9.42 points slightly higher than in the German sample of the C19 ISWS study that was conducted in the beginning of the pandemic [9.25 points, (25)]. This is an important finding showing that the mean level of depressive symptoms remains overall at the same level, because the samples were from the same universities and therefore to some extent comparable. We can thus conclude, that during the long duration of the pandemic the level of depressive symptoms that was measured during the first lockdown did not diminish toward the later phase of the pandemic. Longitudinal studies corroborate this trend: compared to pre-pandemic times, prevalence rates of depressive symptoms during the pandemic seem to have risen and remained high (49).

This pattern also extends to anxiety symptoms (49). In terms of anxiety, we found a prevalence of 31.5%, which is comparable to the pooled prevalence rates of two meta-analyses considering data from university students worldwide [32%, (30), 31%, (50)] and the findings from a German longitudinal study [36%, (49)]. A study conducted in April 2020 involving college students from the US found that anxiety increased due to the COVID-19 pandemic. It showed that university students reported an increased worry about their own health and the health of their loved ones during the COVID-19 pandemic (51). These results are in accordance with our findings that university students who were concerned to get (re-)infected or that someone in their personal network gets severely ill from COVID-19 were at a higher risk for experiencing anxiety. The accumulation of our findings alongside those of other cross-sectional and longitudinal studies contributes to a substantial database, emphasizing the urgency for action and continued monitoring of depressive and anxiety symptoms in university students. It underscores their vulnerability during times of crisis.

A second aim of our study was to examine the factors associated with depressive and anxiety symptoms. Our study showed that a complicated relationship status, the lack of a trusted person and financial difficulties (referring both to the possibility of borrowing money and to difficulties in covering monthly expenses) were associated with depressive and anxiety symptoms. Likewise, university students with pre-existing cardiovascular or multiple health conditions had a higher chance of experiencing mental health problems. Male university students and those of health-related subjects showed fewer depressive symptoms and less anxiety. Moreover, being a first-year university student was found to be a protective factor in regard to depressive symptoms.

In line with the analysis performed by Van de Velde, Buffel (27), we found similar associations with gender, a complicated relationship, financial problems, not having a trusted person and depressive symptoms. However, some findings are not in line with Van de Velde, Buffel (27), and, e.g., was neither age associated in any of our models, nor was migration background associated with depressive symptoms as reported by Van de Velde, Buffel (27). This disparity may be explained by the fact that our sample contained only data from Germany, while the prior analysis was based on an international sample. Variations in cultural backgrounds, support networks, educational environments, and mental health services available to university students in Germany compared to those in the international sample used in the prior analysis could account for this disparity.

Considering the factors associated with depressive symptoms, some previous evidence can be confirmed by our results. Similar to our findings, other studies showed that females (16), those with a lower socio-economic status (15) and fewer social support resources (18) and those with pre-existing other health problems (15) were more likely to experience depressive symptoms. The associations between cardiovascular diseases and mental health problems have also been confirmed in the general population (52). In contrast to other study results (17, 49), university students in our sample who identified as gender diverse did not show a higher chance of depressive symptoms.

In terms of anxiety symptoms, our findings resonate with those of Cao, Fang (26), as we similarly observed associations between anxiety symptoms and factors such as social support and financial stability. Moreover, Cao, Fang (26) identified worry about someone in the network being infected as a contributing factor to anxiety symptoms, a trend echoed in our study. However, while their study highlighted concern about someone being infected, our findings extended this to include specific anxiety regarding severe illness among relatives and personal infection risk.

According to our results, enrolment in a health-related study subject was a protective factor for both depressive and anxiety symptoms. This finding aligns with the results of the meta-analysis by Heumann, Palacio Siebe (4) who found lower prevalence rates for medical students compared to an overall prevalence rate for university students in Germany. As medical students have often been in the research focus so far (53, 54), further research should also consider other student groups to verify our results.

In addition, we found being a fist-year university student to be a protective factor for depressive symptoms. Existing evidence in this regard is mixed. The analysis by Puthran, Zhang (55) e.g., showed that the prevalence of depressive symptoms was highest in first-year medical students and decreased in the following years. According to them, medical school itself could be a stressor for university students, especially in the first year. Tsiouris, Werner (49) in their longitudinal study also found that being a first-year student to be a risk factor for mental burden. Conversely, other longitudinal studies suggest a deterioration in mental health over the course of the study (56) whereas findings from a cross-sectional study showed no difference between in depressive symptoms of first year and advanced students (57).

While several variables exhibited inconsistent associations across models, potentially due to variations in the number of cases included in logistic models, our first model revealed that individuals living alone were more prone to reporting depressive symptoms compared to those living with others. Likewise, Cao, Fang (26) found that living with parents was associated with lower levels of anxiety. However, this association was not uniformly significant across models. According to our analyses, being single was not associated with depressive or anxiety symptoms in our study, but other research suggests it may be linked to mental health: an association between relationship status and depressive symptoms was found by Shah, Mohammad (58) in their survey conducted during the pandemic. Tsiouris, Werner (49) demonstrated a protective effect of having a partner in regard to depressive symptoms and according to their findings, living with others is associated with fewer depressive symptoms and anxiety. People who live alone or are single may feel lonely which may explain the higher likelihood of depressive symptoms (59, 60).

In our study, we found associations with depressive and anxiety symptoms across different levels, both individual and beyond, as outlined by the socio-ecological model (22). To build on these insights, future research should adopt a more comprehensive approach that encompasses all levels of the model, including societal-level factors. This would provide a deeper understanding of how systemic and structural elements influence mental health outcomes, offering a more holistic perspective consistent with the socio-ecological framework.

### 4.1 Strengths and limitations

This multi-center study provides evidence on the prevalence of depressive symptoms and anxiety among university students in Germany and the factors associated with both indicators for mental wellbeing of university students. However, some limitations of the study must be considered. First, it was conducted with a convenience sample from five universities in Germany, meaning that the results are not representative of the German university student population in general. Likewise, more than a quarter of the participants were university students of medicine or health-related subjects, which again limits the generalizability of the results to the entire university student population. The sample was gender imbalanced, and a selection bias cannot be ruled out. Moreover, as this is a cross-sectional study that is largely based upon the C19 ISWS survey and involves a similar, but not the same study population, it is not possible to draw final conclusions about causality or the change in depressive or anxiety symptomatology over the duration of the pandemic. Therefore, we cannot estimate, for example, the onset of mental health problems or after what degree of financial difficulties a significant association between anxiety and depressive symptoms can be found.

In the C19 GSWS survey, self-assessed measures were used, which may have introduced response bias. To mitigate this potential distortion, we collected data through a confidential online survey using established instruments. However, the CES-D 8 is one instrument for assessing depressive symptoms, but it is not validated for university students as well as the higher education context and lacks an established cut-off value. This is why we decided not to conduct further analyses using this instrument. Future studies should examine the validity of cut-off points for the CES-D 8 for this specific target group. Nevertheless, we were able to explore associations based on the PHQ-2 and GAD-2, even though these instruments are short forms to measure depressive and anxiety symptoms with limited internal validity.

The study did not include measurements on psychological stress, which may, especially for university students, also be an important factor in the COVID-19 pandemic. Furthermore, one variable could not be considered in our models as a covariate due to multicollinearity, namely the concern on getting severely ill by (re-)infection with COVID-19.

## 5 Conclusion

This study showed that mental health problems such as anxiety and depressive symptoms were widespread among university students during the pandemic and associated with a variety of factors. The analysis showed that social support factors, financial difficulties and pre-existing health conditions were associated with mental health problems. Participants who were worried about (re-)infection with COVID-19 and those who were (very) worried about someone in their personal network becoming seriously ill with COVID-19 reported more anxiety symptoms. The findings can help to develop specific concepts for prevention and counseling, that also consider the burdens, e.g., financial issues, caused by the COVID pandemic and beyond.

Our study and other research in this area show that university students are a vulnerable group when it comes to mental wellbeing. Long-term studies that continuously report on the mental health of university students are still scarce but should be implemented in the future. For this purpose, it is also important to consider a sample of university students that is representative to the student body in terms of gender distribution and field of study.

In addition, further research should broaden the focus beyond merely assessing associated factors and their relationships with depressive and anxiety symptoms. Future studies should include intervention research that explores effective strategies for promoting and maintaining mental wellbeing among university students. For instance, intervention studies could evaluate various approaches, such as online counseling services and digital mental health tools, to determine their effectiveness in supporting students during times of crisis. The COVID-19 pandemic has underscored the critical need for such tailored online interventions that address the unique mental health challenges faced by university students. By identifying and implementing effective support mechanisms, researchers can help ensure that students’ mental health needs are adequately met during both routine and crisis situations, thereby enhancing overall wellbeing and resilience.

## Data Availability

The datasets presented in this study can be found in online repositories. The names of the repository/repositories and accession number(s) can be found at: Zenodo, https://zenodo.org/doi/10.5281/zenodo.7659845.
